# Chronic Primary Tinnitus: A Management Dilemma

**DOI:** 10.3390/audiolres10020010

**Published:** 2020-11-25

**Authors:** Annanya Soni, Abhishek Dubey

**Affiliations:** Department of ENT, All India Institute of Medical Sciences (AIIMS), Raebareli 229405, India; entdubey@gmail.com

**Keywords:** tinnitus, sound therapy, cognitive behavioral therapy, anticonvulsant, gingko biloba, betahistine, tinnitus retraining therapy, zinc

## Abstract

Tinnitus often described as sound in the ear in absence of any external stimulus. It poses a challenge to the psychological and mental wellbeing of the patient and professional unsatisfaction to the clinician. The patient often an old aged individual usually approaches the outpatient department with various sounds in the ear, making him feel ill or unable to have a sound sleep. The middle-aged patient often complains of professional incapability and lack of concentration due to tinnitus. Despite vast academic research and advances, the efficiency of available treatment is debatable, often compelling the clinician to convey the message that “you may have to learn to live with it”. In the present overview of reviews, we tend to look into the management of tinnitus and present a comprehensive outlook of various evidence-based reviews from Cochrane and augmented with various studies from PubMed.

## 1. Introduction

Tinnitus defined as perception of sound in absence of any external stimuli [1]. Traditionally classified as objective and subjective tinnitus. Objective tinnitus is much less common and is attributed to treatable causes, including vascular lesions, palatal and middle ear myoclonus, patulous eustachian tube etc. However subjective tinnitus is heard only by patient and source of sound is not identifiable [2]. There are no criteria to quantify and qualify such tinnitus and severity depends on patient perception. Primary tinnitus is idiopathic and may be associated with sensorineural hearing loss, whereas secondary tinnitus is associated with other pathology apart from sensorineural hearing loss. Dauman classified tinnitus as normal and pathological on the basis of duration [3]. It can be acute or chronic; chronic tinnitus being uncorrected with treatment when it lasts for more than three months [4,5]. Most cases of chronic tinnitus are associated with some form of hearing loss but severity may not correspond to the degree of hearing loss [6,7]. Some people with normal hearing may present with disturbing tinnitus [8]. More recently, the term HHL has been used to describe patients whose hearing loss is undetectable by pure-tone audiometry between 0.25 and 8 kHz, but report difficulties in understanding speech and difficulties tolerating tinnitus [9,10]. Despite a plethora of new advances and research, etiopathogenesis and treatment of chronic tinnitus is debatable.

Traditionally, chronic primary tinnitus was considered to be a disease of the peripheral auditory system but recent theory suggests central auditory nervous system as the source of tinnitus [11]. There exist multiple levels of involvement underlying the mechanism of tinnitus-1, including peripheral 2, acoustic pathway 3, and supratentorial structures [12,13].

### Clinical Evaluation

Ruling out treatable causes of tinnitus is a must for the successful management. Any pathology in external and middle ear needs to be addressed. History of any other associated symptoms should be elicited. Otoscopic examination to look for pathology in the external ear (cerumen, discharge) and tympanic membrane, middle ear is done. This is to be followed by a detailed audiometric evaluation. Laboratory investigation including thyroid profile, blood sugar, and lipid profile to rule out other comorbidities and their management.

Imaging is required to rule out any tumor in unilateral cases. Contrast enhanced MRI is the imaging of choice with high sensitivity and specificity [14,15].

The use of auditory brainstem response (ABR) for tinnitus diagnosis or as an outcome measure is debatable. ABR has been used to differentiate peripheral and central lesion and to evaluate treatment effect on tinnitus. ABR can detect cochlear synaptopathy resulting in hidden hearing loss in patients with tinnitus with normal hearing [9]. Miloy V et al. in a meta-analysis concluded that the tinnitus group with normal hearing reported longer latency and reduced amplitude of wave I as compared to matched controls [16].

Tinnitus questionnaires are developed to grade the severity of tinnitus and differentiate the distress caused by tinnitus from that because of hearing loss. One such questionnaire is Tinnitus and Hearing Survey (THS), which is a screening tool that includes three scales: A (Tinnitus), B (Hearing), and C (Sound Tolerance). The A and B scales contain four items. The score provides an assessment of difficulties caused by tinnitus and hearing loss separately [17]. The tinnitus handicap inventory (THI) is used as a tool for quantifying the impact of tinnitus on everyday life. It consists of a set of 25 questions [18]. The 25-item Tinnitus Functional Index (TFI) containing eight subscales is a new tinnitus outcome measure that is also sensitive to treatment effects [19]. The 26-item TRQ (tinnitus reaction questionnaire) is used for evaluating the psychological distress of tinnitus sufferers; this questionnaire enables the detection of patients whose psychological distress necessitates rapid intervention. VAS (visual analog scale) is a 10 point scoring for tinnitus loudness measurement.

## 2. Materials and Methods

### 2.1. Design

An overview of systemic reviews was undertaken in accordance with PRISMA (Preferred reporting items for systemic reviews and meta-analysis) guidelines. There was no date restriction in conducting a literature search. CDSR and the PUBMED library were searched using the terms ‘tinnitus, management’. Two authors independently screened all reviews. Any disagreement was resolved by discussion and consensus.

### 2.2. Inclusion and Exclusion Criteria

Systemic reviews and meta-analysis of randomized control trials with evaluated efficacy of interventions for chronic tinnitus were included. Where reviews including same trials were found, we included review with maximum no of trials. Where update is available, we used updated review. Non-English studies were excluded. All reviews included patients aged more than 18 years. Any intervention and comparator (placebo, active) were included. Primary outcome measures (tinnitus loudness, tinnitus specific health related quality of life, tinnitus severity and disability) were used for comparison.

## 3. Results

### 3.1. Identification and Selection of Reviews

**Figure 1:**
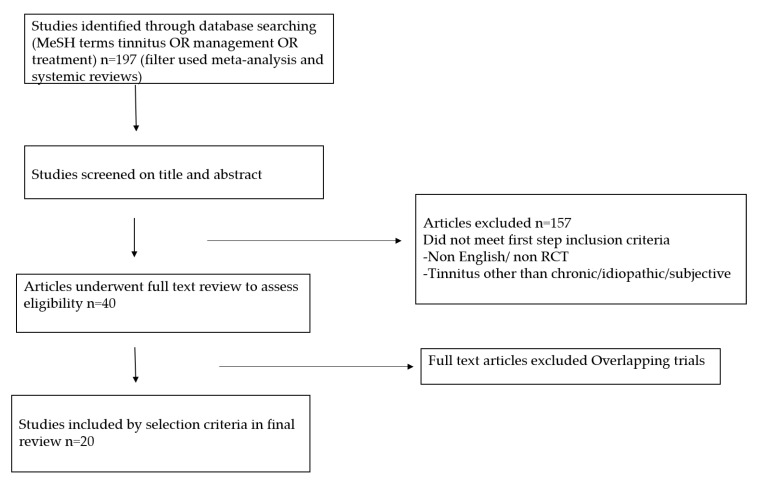
Flow diagram depicting search strategy.

Figure 1 depicts the PRISMA flow chart used to identify and select reviews for inclusion. on searching the various databases with terms (tinnitus, management) we found 197 records. After going through title and abstract 157 were excluded. All the relevant reviews published till date were included. A total of 20 systemic reviews were considered for this overview.

Characteristics of the included reviews. Table 1 provides an insight into the relevant reviews.

Table 2 shows the recommendations for future research and overview authors conclusion. All included reviews recommended the need for further high quality uniform research at a larger sample population.

### 3.2. Treatment

There are several treatment options described in the literature. However, none of these are known to offer a definite relief to patient. Various systemic reviews were taken into account while compiling the treatment options for tinnitus management.

#### 3.2.1. Sound Therapy

Sound therapy consists of either a hearing aid or sound generators maskers or both. There are many theories behind the use of sound therapy. Amplification may change the patient’s focus on alternative sound rather than tinnitus thus reducing stress and anxiety [20,21]. Alternatively, amplified sound leads to Increased neuronal activity thus reducing the audibility and awareness of tinnitus.

According to Sereda et al., hearing aid/maskers/combination device might result in little or no significant benefit in tinnitus severity [22].

Hobson J et al. in a Cochrane review of six trials did not find a strong evidence of the efficacy of sound therapy in tinnitus management. Though absence of conclusive evidence should not be interpreted as evidence of lack of effectiveness, it could be because of lack of quality research [23].

According to Hoare there is limited evidence of the benefit to tinnitus with a hearing aid, but further studies are warranted to reach to any outcome [24].

#### 3.2.2. Cochlear Implant (CI)

Patient with significant hearing loss report improvement in tinnitus after successful cochlear implant surgery [25]. Levy reviewed 17 studies encompassing 247 patients with single sided deafness, he concluded that cochlear implant patients reported significant improvement in tinnitus loudness [26].

#### 3.2.3. Tinnitus Retraining Therapy (TRT)

Jastreboff developed a strategy that combined patient counseling and sound therapy for the management of tinnitus. This was labeled as tinnitus retraining therapy by Hazel. The treatment protocol includes educational counseling about the mechanism of tinnitus and sound therapy is then administered. The therapy is repeated monthly for 3 months, then at 6, 9, 12, 18, and 24 months [1].

According to Philips JS, tinnitus retraining therapy is superior to masking when the TRT protocol is strictly followed [27].

#### 3.2.4. Transcranial Magnetic Stimulation (TMS)

According to pathophysiology of tinnitus, it is more of a central pathology rather than inner ear pathology. Increased neuronal activity in the central auditory system may be responsible for tinnitus. Repetitive transcranial magnetic stimulation (r TMS) can reduce this neuronal activity. Transcranial magnetic stimulation was first used in humans by Barker. Magnetic fields generate an electric current which can penetrate the brain to decrease neuronal activity in central auditory pathway but the results are transient. Repetitive stimulation can outlast the increased neuronal activity [28].

According to the review published by Meng et al., repetitive transcranial magnetic stimulation offers short term benefit in tinnitus but its use in long term is not supported by sufficient data [29].

Another meta-analytic review by Chen et al. is suggestive of beneficial role of central noninvasive brain stimulation (CNBS) in tinnitus severity [30].

#### 3.2.5. Cognitive Behavioral Therapy (CBT)

It includes a combination of behavioral and cognitive therapies to address the psychological aspect of tinnitus rather than auditory. Cognitive therapy works by identifying and altering any maladaptive thinking to more realistic beliefs. Behavioral therapy is targeted to decrease the negative impact of tinnitus on daily life such as continuing the activities which the patient stopped due to fear of tinnitus. We identified four meta-analytic reviews evaluating the role of cognitive behavioral therapy on tinnitus management.

Cochrane meta-analysis by Fuller et al., including 28 studies, showed that there is moderate certainty evidence that CBT is successful in dealing with negative impact of tinnitus on patient life [31]. similarly reviews by Landry et al., Martinez et al. and Hesser et al. are supporting CBT as an effective treatment modality [32,33,34].

#### 3.2.6. Anticonvulsants

The use of anticonvulsant in tinnitus is based on an assumption that tinnitus is caused by central neuronal hyperactivity.

There are three proposed mechanisms for their action:

- Increasing the inhibitory neurotransmitter GABA
- Decreasing the excitatory glutamate transmission
- Blocking the sodium channels

Meta-analysis of seven studies including four anticonvulsants (gabapentin, carbamazepine, lamotrigine, and flunarizine) showed no evidence in having a large positive effect on tinnitus. Additionally, 18% of patients showed some form of side effect from the drugs [35].

#### 3.2.7. Antidepressant and Anxiolytic

The mechanism of action of antidepressant drugs on tinnitus is debatable. It may affect the psychological status of the patient or directly affect central auditory pathway [36]. The most commonly used drugs are tricyclic antidepressant (TCA) and selective serotonin receptor inhibitor (SSRI). Cochrane meta-analysis, including six trials, revealed no statistically significant evidence of efficacy of antidepressant in the treatment of tinnitus [37].

Routine use of anxiolytics is not included in AAO-HNSF guidelines. It may benefit patient with predominant anxiety issues [38].

#### 3.2.8. Gingko Biloba

Proposed mechanisms of action of gingko biloba are (1) Increased blood flow by altering the tone of blood vessels [39]. (2) Antagonism of platelet activating factor thus preventing hypoxic brain injury [40,41]. (3) Prevents free radical mediated injury.

There are various studies that support the use of gingko biloba in tinnitus, but only one trial was found to be of statistically significant evidence [42]. As per Cochrane meta-analysis of four trials by Hilton MP et al., there were limited evidence for proven benefit of gingko biloba in patients where tinnitus was the primary complaint [43].

#### 3.2.9. Zinc Supplementation

Glutamate is a neurotransmitter in inner hair cells [44]. Zinc is presumed to have modulating effects on glutamatergic action in the central auditory pathways. Additionally, zinc is also presumed to exert antioxidant effects on the cochlea. Both of these mechanisms could be related to the improvement of tinnitus in some patients. Systematic administration of zinc has therefore been tested as an alternative treatment for this disorder by several investigators. Person et al. did not find any solid evidence of beneficial effect of zinc on tinnitus management on the basis of systematic review of three trials [45].

#### 3.2.10. Betahistine

Rational for use of Betahistine in tinnitus treatment lies in the theory that changes in endolymphatic pressure may be accountable for tinnitus and hearing loss, Betahistine reduces endolymphatic pressure through vasodilation of the vessels of the of the inner ear [46]. It is more likely to be effective in cases where a non-specific tinnitus is associated with hearing difficulties (or vertigo). According to a meta-analysis of five trials by Wegner et al., there was no evidence of benefit of Betahistine on chronic tinnitus over placebo [47].

#### 3.2.11. Hyperbaric Oxygen Therapy (HBOT)

Hearing loss and tinnitus may result from hypoxic injury to cochlear apparatus, and hyperbaric oxygen therapy may improve this injury. It involves therapeutic administration of 100% oxygen at high pressure. Cochrane review of seven studies found no evidence of any beneficial effect of hyperbaric oxygen therapy on chronic tinnitus [48].

#### 3.2.12. Educational Counseling

It is aimed to promote self-control and acceptance of tinnitus symptoms. Xiang et al. did a meta-analysis to see the effect of educational counselling alone compared with other psychological treatments, he found that educational counselling is helpful in tinnitus patients, and a cost effective treatment [49].

#### 3.2.13. Acupuncture

Acupuncture is a traditional treatment modality used in Chinese culture. The mechanism of action is poorly understood, neuromodulation of the auditory pathway is a possible mechanism. Huang reviewed eight studies involving 504 participants. Acupuncture had a positive effect on tinnitus-related quality of life though no effect was observed on tinnitus loudness [50].

Duckert et al. demonstrated the importance of placebo effect in tinnitus management [51]. Surgical treatment is targeted to the underlying pathology in secondary tinnitus and hardly plays any role in the management of primary tinnitus in current scenario.

#### 3.2.14. Future Therapies

As tinnitus is usually associated with underlying sensorineural hearing loss, any treatment which improves hearing is likely to reduce tinnitus. Genetic therapy holds a promising outcome by promoting regeneration of cochlear hair cells [52]. Another genetic therapy using a viral vector inhibition of neural activity in targeted regions of central auditory pathways have the potential to suppress tinnitus [53]. Neramexane, a NMDA antagonist presently under phase III trial blocks nicotinic cholinergic receptors in the inner ear [54,55]. Lidocaine is an antiarrythmic drug whose potential use in tinnitus management is being studied. Lidocaine is believed to affect the central pathway of tinnitus. Commercially available transdermal lidocaine patches may improve tinnitus [56].

Vesitipitant is a novel antagonist of the neurkinin-1 receptor which binds substance P. Neurokinin receptors are present in the inner ear, representing a potential therapeutic target for tinnitus [57]. Vestipitant and the combination of vestipitant and paroxetine are currently undergoing Phase 2 clinical trials for the treatment of tinnitus.

## 4. Conclusions

Treatment of chronic tinnitus is directed more toward control rather than cure. In most cases, it is likely to persist. Our target should be to improve quality of life and reduce the effects of tinnitus on mental and physical wellbeing of the patient. The clinician should select a specific treatment with caution and customize the treatment plan on a patient to patient basis, rather than one for all treatment policy. Further robust trials following strict methodology are required to prove the efficacy of treatments available for tinnitus management.

## Figures and Tables

**Table 1:** Characteristics of included studies.

s.n	Study	No of RCT/ Pt	Intervention	Comparator	Outcome (95% CI)	Level of Evidence
1.	Fuller et al.	28/2733	CBT	No intervention (10 RCT/ 537)	SMD 0.56 lower (0.83 lower to 0.30)	low
				Audiological care (3 RCT/ 430)	MD 5.65 lower (9.79 lower to 1.5 lower)	moderate
				TRT (1 RCT/42)	MD 15.79 lower (27.91 lower to 3.67)	low
				Active control (12 RCT/966)	SMD 0.30 lower (0.55 lower to. 0.05)	low
2.	Martinez et al.	8/468	CBT	no intervention(6RCT/354)	SMD 0.24 (−0.02 to 0.051)	-
				Yoga/education (4/164)	SMD 0.1 (−0.22 to 0.42)	-
3.	Landry et al.	11/1111	CBT	no intervention (11RCT/1111)	SMD 1.46 (0.67 to 2.24)	low
4.	Hesser et al.	15/1091	CBT	no intervention (14RCT)	SMD 0.70 (0.56 to 0.84	low
				Education /TRT/yoga (5RCT)	SMD 0.44 (0.16 to 0.72)	low
5.	Baldo et al.	6/610	TCA	different trials could not be combined		
6.	Bennett et al.	2/83	HBOT	sham / pharmacological therapy	RR 0.68 (0.32 to 1.42)	moderate
7.	Hilton et al.	4/1543	ginkgobiloba	placebo, different trials could not be combined		low
8.	Hoare et al.	1/91	hearing aids	sound generators	MD −0.90 (−7.92 to 6.12)	moderate
9.	Hobson et al.	6/533	sound therapy	different trials could not be combined		
10.	Hoekstra et al.	7/453	Gabapentin	Placebo	SMD 0.07 (−0.26 to 0.40)	low
11.	Huang et al.	8/504	acupuncture	sham acupuncture	MD −2.18 (−4.89 to 0.53)	low
				Drug therapy	MD −1.33 (−2.01 to −0.65)	low
12.	Levy et al.	17/247	CI	Pt prior to intervention	VAS MD −4.6 (−6 to −3.3)	low
					THI MD −35.4 (−55.8 to −15)	
13.	Meng et al.	5/233	r TMS	sham r TMS	RR 12 (1.76 to 81.74)	moderate
14.	Dong et al.	10/567	r TMS	sham r TMS	VAS SMD −0.28 (−0.59 to 0.02)	moderate
					THI SMD −0.04 (−0.23 to 0.16)	
15.	Chen et al.	32/1458	central NIBS	Sham control	SMD −1.89 (−3 to −0.78)	moderate
16.	Person et al.	3/209	zinc	different trials could not be combined		very low
17.	Philips et al.	1/123	TRT	tinnitus masking		low
18.	Sereda et al.	8/590	sound therapy	different trials could not be combined		low
19.	Xiang et al.	9/582	educational counseling	other psychological and combination therapy	MD 3.59 (−0.56 to 7.74)	moderate
20.	Wegner et al.	5/303	Betahistine	Placebo	MD −0.16 (−1.01 to 0.70	very low

SMD-standardized mean difference; MD-mean difference; RR-relative risk; CI-confidence interval.

**Table 2:** Interpretation of results by original and overview authors.

Review	Primary Outcome	Review Author Conclusion	Overview Author Conclusion
Fuller et al.	THI score	CBT may be effective in reducing the negative impact on quality of life	CBT is a cost effective measure, helpful in alleviating tinnitus associated negative impact on patient’s life, though it may not reduce the tinnitus volume.
Martin et al.	Tinnitus loudness (VAS)	CBT is unlikely to be effective in improving subjective tinnitus loudness but effective for improving quality of life	
Landry et al.	QOL	CBT had larger effect sizes on tinnitus-related QOL	
Hesser et al.	QOL	CBT is an effective treatment in reducing annoyance and distress associated with tinnitus	
Baldo et al.	Tinnitus severity and disability	No evidence that TCA are effective in management of tinnitus	Further trials with uniform robust methodology are required to compare the results and derive any conclusions
Bennet et al.	Relief of tinnitus	No suggestion that HBOT improves chronic tinnitus	Only two trials report outcome for chronic tinnitus (demand a cautious interpretation owing to low no of patients)
Hilton et al.	Tinnitus loudness (VAS), Tinnitus associated QOL	Not demonstrated to be effective for primary tinnitus	Further high quality trials with uniform methodology are required
Hoare et al.	Tinnitus severity	No evidence to support or refute the use of hearing aids	The benefit of sound therapy alone in the treatment of tinnitus is unproven. Use of hearing aid improves the hearing handicap and quality of life. Further trials are required to support the efficacy of hearing aids in tinnitus handicap.
Hobson et al.	Tinnitus loudness, QOL	No evidence that a significant change in loudness of tinnitus can be achieved by sound generator as solo intervention	Further trials with uniform methodology are required for analysis
Hoekstra et al.	Tinnitus specific health related QOL	Do not show a beneficial effect on tinnitus (significant risk of bias)	Further methodological trials of high quality are required
Huang et al.	Tinnitus loudness (VAS)	Did not have significant impact on tinnitus loudness but did improve tinnitus specific health related QOL	Further high quality studies are required to confirm the efficacy of acupuncture
Levy et al.	Tinnitus specific health related QOL, Tinnitus loudness (VAS)	Significant reduction in THI and VAS score	Cochlear implant is an effective treatment strategy in patients with severe to profound hearing loss. Further trials of high quality are needed to support their use for tinnitus.
Person et al.	Tinnitus severity and disability (THI)	No evidence that the use of oral zinc improve tinnitus	Further trials with uniform methodology are warranted to analyse the results
Philips et al.	Tinnitus severity and disability (THI)	TRT is much more effective than tinnitus masking	Single low quality RCT. Further high quality studies with robust methodology are required using protocols as proposed by Jastreboff 1999
Dang et al.	Tinnitus severity and disability (THI), tinnitus loudness	rTMS not effective in treating chronic tinnitus	Further studies with uniform protocols are needed to explore the potential benefits of r TMS in chronic tinnitus
Chen et al.	Tinnitus severity	Central noninvasive brain stimulation is associated with improvement in tinnitus severity	
Meng et al.	Tinnitus severity and disability (THI)	Limited support for the efficacy of rTMS in treating patients with tinnitus	
Xiang et al.	Tinnitus severity	Educational counseling alone is helpful in improving tinnitus and has same effect as other psychological and combined treatment	It is a cost effective measure useful in decreasing tinnitus-related negative impact on the patient’s life.
Wegner et al.	Tinnitus loudness	Absence of any evidence suggestive of beneficial effect of betahistine on tinnitus	High quality trials are required to derive any conclusion
